# Phylogeographic and Host Interface Analyses Reveal the Evolutionary Dynamics of SAT3 Foot-And-Mouth Disease Virus

**DOI:** 10.3390/v17121641

**Published:** 2025-12-18

**Authors:** Shuang Zhang, Jianing Lv, Yao Lin, Rong Chai, Jiaxi Liang, Yan Su, Zhuo Tian, Hanyu Guo, Fuyun Chen, Guanying Ni, Gang Wang, Chunmei Song, Baoping Li, Qiqi Wang, Sen Zhao, Qixin Huang, Xuejun Ji, Jieji Duo, Fengjun Bai, Jin Li, Shuo Chen, Xueying Pan, Qin La, Zhong Hong, Xiaolong Wang

**Affiliations:** 1 Key Laboratory of Wildlife Diseases and Biosecurity Management of Heilongjiang Province and Sino-Ethiopian Wildlife Disease Research Joint Laboratory, Northeast Forestry University, Harbin 150040, China; zhangshuang@nefu.edu.cn (S.Z.); liangjiaxi2022@163.com (J.L.); 814268442@qq.com (Y.S.); roseiny6206@163.com (Z.T.);; 2 The Haixi Prefecture Center for Animal Disease Control and Prevention, Delingha 817000, China; 3 The Haixi Extension Service Center for Agricultural and Animal Husbandry Technology, Delingha 817000, China; 4 The Haixi Forest Pest Control and Quarantine Center, Delingha 817000, China; 5 General Affairs Department, attached to the Administrative Committee of Huzhu Green Industry Park, Haidong Industrial Park, Haidong 810600, China; 6 Delingha Center for Animal Disease Control and Prevention, Delingha 817000, China; 7 Gahai Township Animal Husbandry and Veterinary Station, Delingha 817000, China

**Keywords:** foot-and-mouth disease, SAT3, phylogeographic, evolutionary, cross-species transmission

## Abstract

Foot-and-mouth disease virus (FMDV) serotype SAT3 is a rarely studied serotype primarily circulating in southern Africa, with African buffalo (*Syncerus caffer*) serving as its key reservoir. In this study, we performed a comprehensive phylogenetic and phylodynamic analysis of SAT3 based on 81 full-length VP1 gene sequences collected between 1934 and 2018. Maximum likelihood and Bayesian analyses revealed five distinct topotypes, each with clear geographic and host associations. Notably, topotypes I, II and III were observed in both African buffalo and cattle (*Bos taurus*), while topotype IV appeared restricted to African buffalo. Likelihood mapping indicated moderate to strong phylogenetic signal, and the mean substitution rate was estimated at 3.709 × 10^−3^ substitutions/site/year under a relaxed molecular clock. The time to the most recent common ancestor (TMRCA) was traced back to 1875. Discrete phylogeographic reconstruction identified Zimbabwe as a major center, with multiple supported cross-border transmission routes. Host transition analysis further confirmed strong directional flow from buffalo to cattle (BF = 1631.09, pp = 1.0), highlighting the wildlife–livestock interface as a key driver of SAT3 persistence. Together, these results underscore the evolutionary complexity of SAT3 and the importance of integrating molecular epidemiology, spatial modeling, and host ecology to inform FMD control strategies in endemic regions.

## 1. Introduction

Foot-and-mouth disease (FMD) is a highly contagious, acute viral disease affecting cloven-hoofed animals, including cattle, pigs, sheep, goats, and numerous wild ungulates [1,2]. The etiological agent, foot-and-mouth disease virus (FMDV), is a positive-sense, single-strand RNA virus classified within the genus *Aphthovirus* of the family *Picornaviridae* [3]. Based on serological and genetic distinctions, FMDV is classified into seven serotypes: O, A, C, Asia1, and Southern African Territories (SAT) types 1–3, no immunological cross-reactivity (or cross-protection) is observed [4,5].

Foot-and-mouth disease (FMD) imposes substantial economic burdens in both endemic and previously disease-free regions. Beyond the direct effects on livestock productivity—such as reduced milk yield, diminished traction capacity, and restrictions on animal movement and trade—FMD control efforts also require sustained financial investment. In endemic areas, the combined costs of production losses and vaccination programs are estimated to range between USD 650 million and 21 billion annually [6]. Moreover, outbreaks occurring in FMD-free countries can result in yearly losses exceeding USD 150 million, primarily due to emergency response measures and trade disruptions [5]. Although adult animals often recover from infection, young animals may develop acute myocarditis, leading to high mortality rates, with some viral strains causing case fatality rates approaching 100%.

In the 1940s, three additional serotypes (SAT1, SAT2, SAT3) were identified in southern Africa [7,8]. SAT3, the rarest and least studied of the SAT serotypes, has been sporadically reported in wildlife and livestock across South Africa, Zimbabwe, Mozambique, Zambia, Namibia, Botswana, Malawi, and Uganda. Historical outbreaks have been documented across multiple decades, including a notable epidemic in Kruger National Park during 1958–1959 involving blue wildebeest (*Connochaetes taurinus*), kudu (*Tragelaphus strepsiceros*), and sable antelope (*Hippotragus niger*) [9]. Additional SAT3 outbreaks were reported in Mozambique, as well as in South Africa in 2002 (Phalaborwa, affecting buffalo), 2006 (Thulamela), and 2008 (Pafuri, affecting impala) [9]. Notable outbreaks include a 9-month epidemic in Giyani (Limpopo Province) during 1979/1980 [10], as well as more recent reports in South Africa (2021–2024), Namibia (2011), and Zambia (2015, 2017) (World Organization for Animal Health). These events suggest a persistent, yet poorly understood pattern of SAT3 circulation and cross-border spread. Importantly, wild hosts such as African buffalo (*Syncerus caffer*) play a critical role in the maintenance and transmission of SAT serotypes [11,12]. Their ability to harbor the virus asymptomatically over long periods significantly complicates FMD control efforts in regions where wildlife and livestock share ecological interfaces.

The FMDV genome is approximately 8.5 kb in length and encodes a polyprotein that is cleaved into 12 proteins, including four structural (VP1–VP4) and eight non-structural proteins (e.g., 3ABC, 3Dpol) [13]. Among these, the VP1 coding region is highly variable and serves as a key molecular marker for phylogenetic analysis and serotype discrimination [14]. It displays the lowest nucleotide and amino acid sequence conservation among the structural proteins, with approximately 50–70% nucleotide identity between serotypes. Monitoring the evolutionary dynamics of FMDV is critical for understanding viral spread, anticipating outbreaks, and designing effective vaccines and control strategies. Phylogenetic analyses based on VP1 sequences have been widely used to track viral diversification and infer transmission events [15,16,17].

However, studies focusing on SAT3 remain scarce, limiting our understanding of its evolutionary trajectory, host associations, and geographic diffusion patterns. To address this gap, the present study aims to investigate the molecular evolution and spatial dynamics of the FMDV SAT3 serotype. Using Bayesian phylogenetic analysis based on VP1 sequences, we reconstructed a maximum clade credibility (MCC) tree and applied discrete trait models to infer geographic and host transitions. By integrating sequence data, spatiotemporal modeling, and Bayes factor-supported migration inference, this study seeks to elucidate SAT3’s dispersal history and transmission hotspots. The insights gained are expected to support the development of more accurate prevention strategies, enhance SAT3-specific vaccine matching, and contribute to broader efforts in transboundary animal disease surveillance and control.

## 2. Method

### 2.1. Sequence Dataset Preparation

Metadata including sampling year, host species, and country of origin were collected for each sequence. To avoid redundancy, only one representative sequence was retained when multiple entries showed 100% nucleotide identity, isolated in the same year and host. After downloading and filtering the dataset from GenBank (NCBI: http://www.ncbi.nlm.nih.gov (accessed on 19 August 2025)), a total of 81 VP1 gene sequences of FMDV serotype SAT3 were retained, covering sampling years between 1934 and 2018 (Supplementary Table S1).

### 2.2. Sequence Alignment and Recombination Screening

Multiple sequence alignment was performed using MAFFT v7.222 with default parameters [18]. Recombination was assessed using a two-step validation approach: first, potential recombination signals were evaluated using RDP v4.95 and SplitsTree v4.14.6. Within RDP [19], seven detection algorithms—RDP, GENECONV, BOOTSCAN, MAXCHI, CHIMAERA, SISCAN, and 3SEQ—were employed under Bonferroni correction with a significance threshold of *p* < 0.01. Sequences were considered recombinant if at least four of the seven algorithms detected recombination events with *p* < 1 × 10^−6^. These candidate recombinants were excluded from further analysis. Non-recombinant sequences were then assessed using SplitsTree with the Neighbor-Net method to visualize network topologies and further validate absence of recombination. A total of 75 non-recombinant VP1 sequences from five countries were retained for subsequent analyses.

### 2.3. Saturation and Phylogenetic Signal Testing

To evaluate substitution saturation, Xia’s test was performed using DAMBE v7 [20]. Sequences with observed index of substitution saturation (Iss) values significantly lower than the critical Iss.c (with *p* < 0.05) were considered unsaturated and retained for tree reconstruction.

### 2.4. Phylogenetic Reconstruction and Clade Assignment

ModelFinder [21] was used to determine the best-fit nucleotide substitution model. Likelihood mapping was conducted using TREE-PUZZLE v5.3 [22] to assess phylogenetic signal based on 10,000 randomly selected quartets. Maximum likelihood (ML) trees were constructed using IQ-TREE v.1.6.12 [23] under the GTR + G + I model, with 1000 bootstrap replicates to estimate node support. The resulting ML tree was visualized in FigTree v1.4.3 and annotated with country and host information using Evolview v2. Clades were assigned based on two criteria: (1) bootstrap support ≥100%, and (2) inclusion of at least two sequences per clade.

### 2.5. Genetic Distance Analysis

To quantify within- and between-topotype divergence, evolutionary distances were calculated using the Maximum Composite Likelihood method implemented in MEGA v7.0.14 [24], incorporating gamma-distributed rate variation among sites [25].

### 2.6. Temporal Signal and Molecular Clock Estimation

Temporal signal was evaluated using TempEst v1.5 [26] in BETS. Bayesian phylogenetic inference was carried out using BEAST v1.10.4 [27] with BEAGLE [28] library acceleration. Evolutionary rates and time to most recent common ancestor (TMRCA) were estimated under the GTR + G + I substitution model. Six clock-prior model combinations (strict/relaxed clocks × constant/exponential/Bayesian Skyline coalescent priors) were tested, and marginal likelihoods were compared via Path Sampling and Stepping-Stone Sampling to select the best-fit model [29,30]. The best-fitting model was identified as the uncorrelated lognormal relaxed clock combined with the Bayesian Skyline coalescent prior, based on the highest marginal likelihood score. MCMC chains were run for 1 × 10^8^ generations, with 10% burn-in and sampling every 100,000 steps. Convergence was assessed in Tracer v1.7 [31] based on effective sample sizes (ESS > 200). The maximum clade credibility (MCC) tree was generated using TreeAnnotator and visualized in Evolview v2. Bayesian Skyline plots were used to infer population dynamics of SAT3 over time.

### 2.7. Spatiotemporal Spread and Host Transition Analyses

To reconstruct geographic dispersal, discrete-trait Bayesian phylogeographic analysis was performed in BEAST using country and host as traits. All discrete trait reconstructions were performed using a fixed empirical tree, previously estimated under a non-reversible asymmetric substitution model with Bayesian Stochastic Search Variable Selection (BSSVS). The BSSVS framework restricts the number of transition rates to only those that contribute significantly to the phylogenetic diffusion process, thereby improving model efficiency and interpretability. Well-supported state transitions were identified with posterior probability ≥0.95. BF values > 3 were considered significant and used to identify high-confidence migration routes, visualized with SPREAD [32] and Google Earth. Additionally, symmetric continuous-time Markov chain (CTMC) models were used to estimate host-to-host and country-level transmission probabilities, with 95% highest posterior density intervals reported.

### 2.8. Selection Pressure and Co-Evolutionary Signal Analysis

Codon-level selection analysis was performed on the VP1 dataset using the Datamonkey [33] platform (http://www.datamonkey.org). Selection was inferred from methods such as SLAC, FEL, MEME, and FUBAR, with a *p*-value < 0.05 considered significant [34,35]. Bayesian Graphical Models (BGMs) were used to detect co-evolutionary interactions among codon sites, with posterior probabilities ≥ 0.95 indicating statistically supported site-pair associations.

The ratio of nonsynonymous to synonymous substitution rates (dN/dS) was used to classify selective pressures as positive (dN/dS > 1), neutral (dN = dS), or purifying (dN/dS < 1) [36].

## 3. Results

### 3.1. Likelihood Mapping and Phylogenetic Analysis

**Figure 1:**
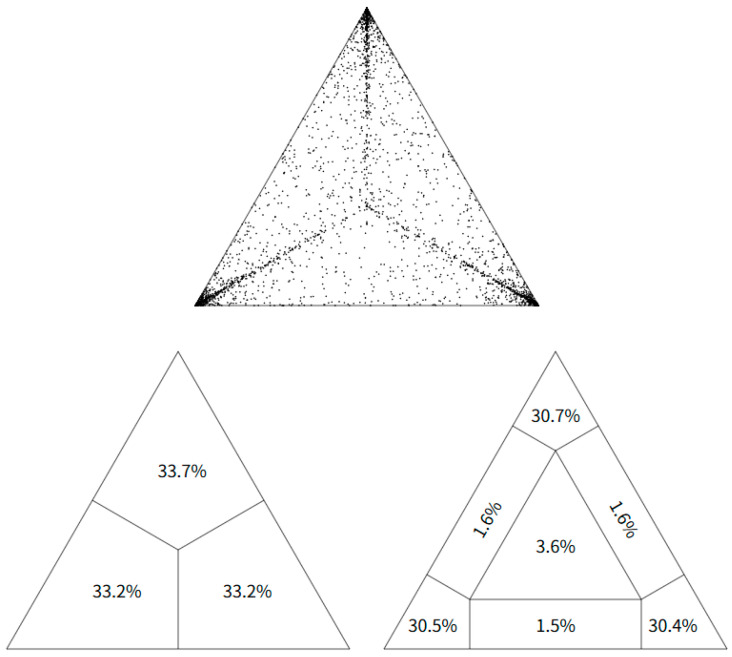
Likelihood mapping analysis of the VP1 gene sequences from SAT3 FMDV revealed sufficient phylogenetic signal for tree reconstruction. Analysis was based on 10,000 randomly selected sequence quartets using TREE-PUZZLE. Only 3.6% of quartets fell into the central unresolved region, indicating moderate-to-strong phylogenetic signal supporting robust downstream evolutionary inference.

Likelihood mapping analysis of the VP1 gene sequences showed that 33.7% of quartets were fully resolved, 33.2% were unresolved, and 33.2% were partially resolved. Only 3.6% of quartets fell into the central star-like region, indicating a moderate to high phylogenetic signal suitable for further phylogenetic inference (Figure 1).

**Figure 2:**
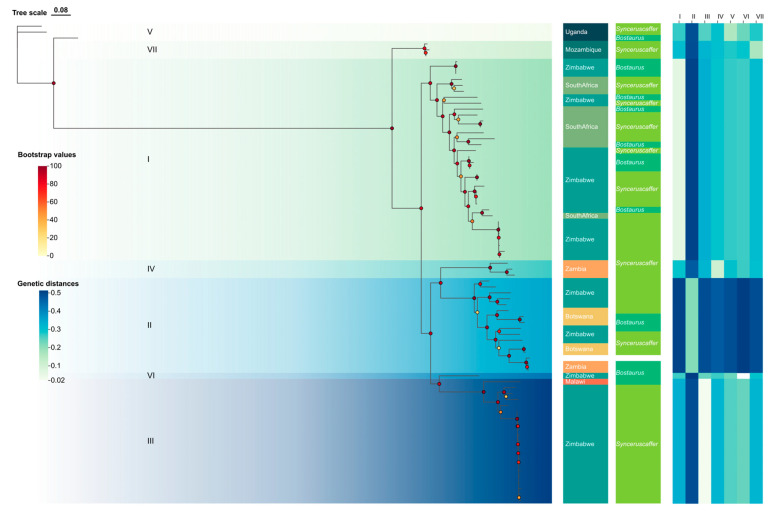
Maximum likelihood phylogeny of 81 non-recombinant SAT3 VP1 sequences revealed seven distinct topotypes with host and geographic associations. The circular tree showed seven well-supported topotypes (topotypes I–VII) with country and host annotations. Bootstrap values ≥ 95% are shown. Sequences from South Africa, Zimbabwe, and Zambia are widely distributed, while Botswana and Mozambique are more topotype-restricted.

We adopted the WRLFMD (FMDbase) scheme for SAT3 and treated eight topotypes (I–VIII) as the reference classification based on >18% VP1 nucleotide divergence and clustering to WRLFMD prototypes. In our dataset, sequences mapped to seven of these topotypes (I–VII) (Table 1); topotype VIII was not represented due to the absence of corresponding sequences in our sample set. Sequences from South Africa, Zambia, and Zimbabwe were distributed across multiple topotypes, while Botswana and Mozambique showed more restricted representation (Figure 2).

Intergroup genetic distances ranged from 0.23 to 0.51. The smallest distance was observed between lineages V and VI (0.2343), followed by VI–III (0.2403) and III–V (0.2551), suggesting that lineages III, V, VI form a closely related cluster. Another group comprising lineages V, VI and VII showed intermediate distances (≈0.23–0.328), reflecting a moderately close relationship within this set. In contrast, the greatest divergences were observed between II–VI (0.5133), I–II (0.5052), and II–VII (0.492), highlighting substantial genetic differentiation between clusters.

Topotypes I, II, III and V include both buffalo and cattle sequences, while IV and VII are exclusive to buffalo, indicating topotype-specific host partitioning.

### 3.2. Demographic and Temporal Analysis

**Figure 3:**
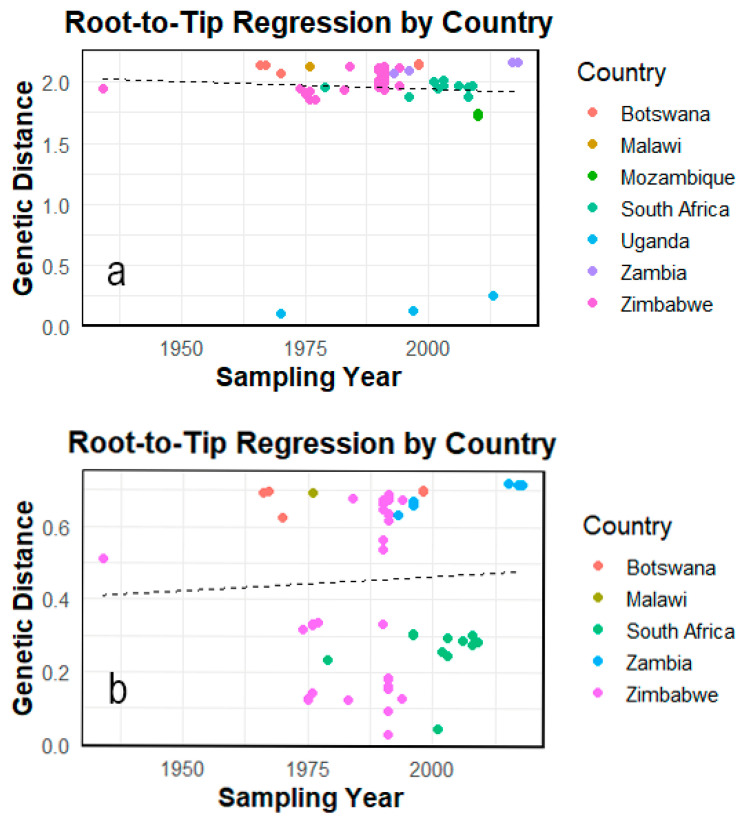
Root-to-tip regression plot showing the temporal signal of the VP1 dataset. (**a**) Full dataset (*n* = 81); (**b**) filtered dataset (*n* = 75). Analyses were performed in TempEst with best-fit root placement. The fitted linear trend (dashed) showed a positive correlation between sampling year and root-to-tip distance, indicating temporal signal in both datasets and supporting the use of relaxed molecular-clock models for evolutionary rate estimation and TMRCA inference.

**Figure 4:**
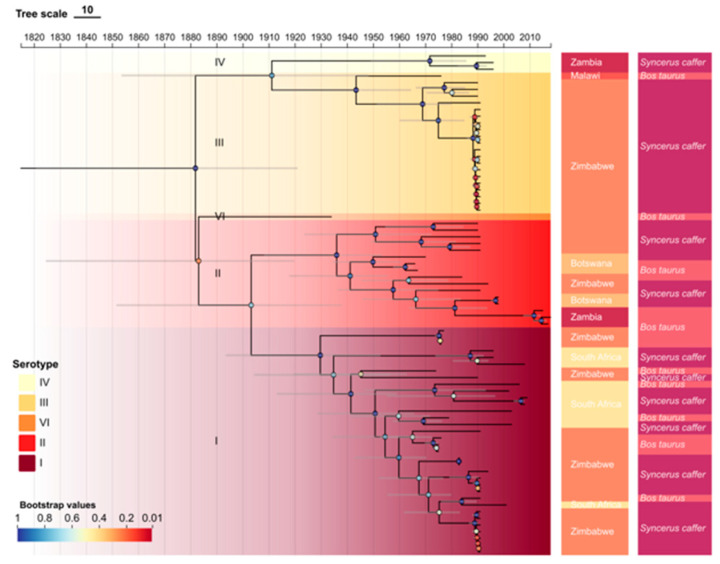
Time-scaled MCC phylogeographic tree of FMDV SAT3 (*n* = 75) in southern Africa. Branches are colored by posterior probabilities values (red = high support), and 95% highest posterior density (HPD) intervals for node dates are indicated by horizontal bars. Five topotypes (I–V) are highlighted by shaded backgrounds. Sampling countries are indicated by side color strips, and host species are marked by symbols: *Bos taurus* (blue squares) and *Syncerus caffer* (orange circles).

**Figure 5:**
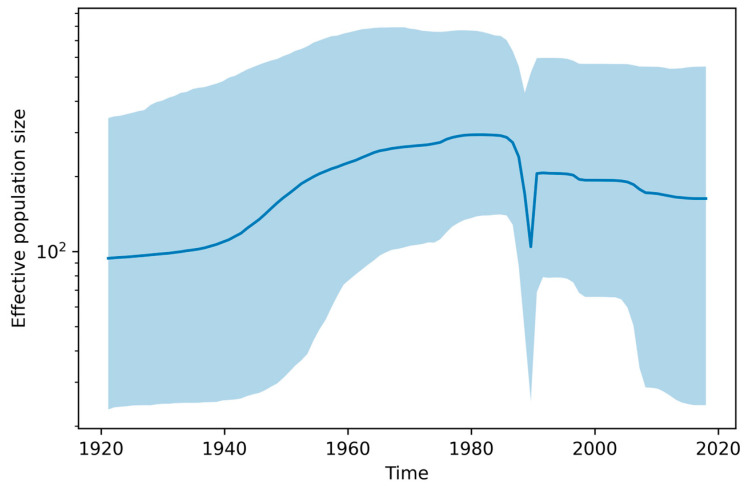
Bayesian Skyline Plot of SAT3 VP1 gene showing effective population size over time. Estimates are based on 75 VP1 sequences under a relaxed lognormal molecular clock with Bayesian Skyline coalescent prior.

Root-to-tip regression of VP1 sequences demonstrated a clear temporal signal in both datasets, without significant outliers, supporting clock-like evolution (Figure 3a,b). Notably, the full dataset (*n* = 81) contained sequences with negative temporal signal; therefore, only the filtered dataset (*n* = 75) showing a positive temporal signal was retained for subsequent molecular clock analyses. The evolutionary rate under the uncorrelated lognormal relaxed clock was estimated at 3.709 × 10^−3^ substitutions/site/year (95% HPD: 1.9141 × 10^−3^ to 5.9644 × 10^−3^). The time to the most recent common ancestor (TMRCA) of SAT3 was inferred to be in 1875 (95% HPD: 1815–1921) (Figure 4), with Zimbabwe identified as the most likely root location. Bayesian skyline plot analysis showed a rise in viral population size around 1940, peaking in 1985, followed by a sharp drop by 1990, then stabilizing with another decline starting from 1993 (Figure 5).

### 3.3. Geographic Dispersal Analysis

**Figure 6:**
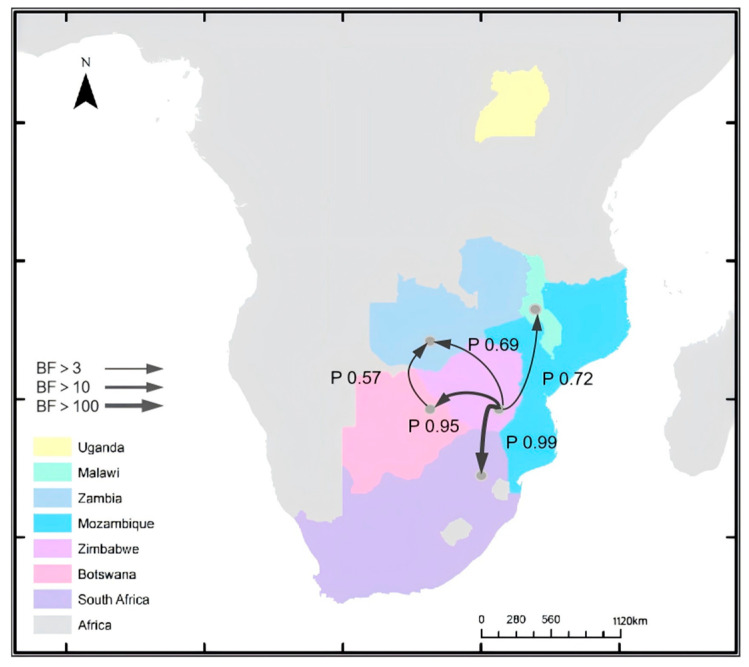
Network diagram of SAT3 geographic migration routes. Phylogeographic reconstruction of the spatiotemporal spread of SAT3 foot-and-mouth disease virus (FMDV) in southern Africa. Arrows represent the most supported viral migration pathways between countries, with arrow thickness indicating the strength of support based on Bayes factor (BF > 3, BF > 10, and BF > 100). Colors indicate different countries: Uganda (yellow), Malawi (green), Zambia (blue), Mozambique (cyan), Zimbabwe (light violet), Botswana (pink), and South Africa (purple), with the background in gray representing Africa.

Bayesian discrete phylogeographic analysis revealed multiple statistically supported cross-border transmission routes of FMDV serotype SAT3 in southern Africa (Figure 6). In total, five inter-country migration events with Bayes factor (BF) > 3 were identified and visualized as a transmission network. Among these, two routes exhibited very strong support, with BF values exceeding 10 and 100, respectively. The most strongly supported pathway was from Zimbabwe to South Africa (BF > 100; posterior probability = 0.99), indicating a highly credible transmission direction. Another strongly supported route was from Zimbabwe to Botswana (BF > 100; posterior probability = 0.95). Additional routes included transmissions from Zimbabwe to Malawi (posterior probability = 0.72) and from Zimbabwe to Zambia (posterior probability = 0.69), both with moderate posterior support. A weaker but detectable pathway was suggested from Botswana to Zambia (posterior probability = 0.57). Collectively, these findings highlight Zimbabwe and Zambia as central nodes in the SAT3 transmission network, with South Africa emerging as a major recipient of viral introductions.

### 3.4. Host-Associated Transmission Analysis

This table (Table 2) summarizes the mean transition rates (per year), Bayes factors (BF), and posterior probabilities for directional transmission events between African buffalo and cattle. Rates were estimated under a Bayesian stochastic search variable selection (BSSVS) model using BEAST. Effective sample sizes (ESS) for the rate estimates are shown in parentheses. Transitions with BF > 3 are considered statistically supported. The most frequent transition occurred from African buffalo to cattle, with a mean rate of 1.08. Bayesian factor analysis revealed strong support (BF = 1631.09, PP = 1) for the African buffalo to cattle direction.

### 3.5. Selection Pressure and Co-Evolutionary Signals

**Figure 7:**
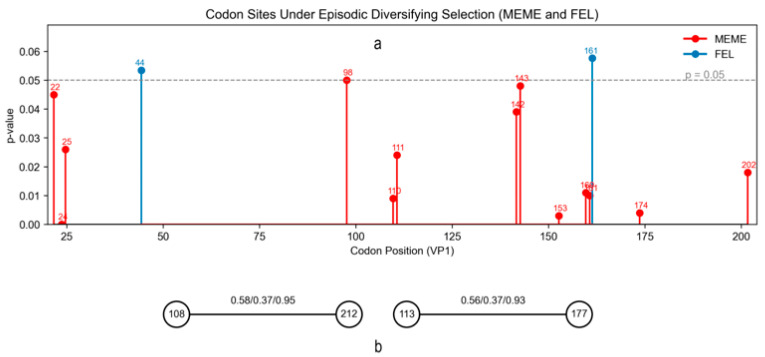
Results of codon-level selection and co-evolutionary analysis of the VP1 coding region. (**a**) Codon sites under episodic diversifying selection identified by MEME (red) and FEL (blue). The y-axis shows the *p*-values, with the dashed line indicating the significance threshold (*p* = 0.05). MEME detected 13 codons (22, 24, 25, 98, 110, 111, 142, 143, 153, 160, 161, 174 and 202) under significant selection, whereas FEL detected no sites that passed the strict threshold but showed borderline signals at codons 44 and 161 (*p* slightly > 0.05). (**b**) Co-evolving codon pairs inferred using the Bayesian Graphical Model (BGM) approach. Each node represents a codon site, and edges denote co-evolutionary relationships with associated posterior probabilities. Edge labels indicate the estimated directional and bidirectional probabilities (*P*[Site1→Site2]/*P*[Site2→Site1]/*P*[Site1↔Site2]), highlighting pairs of codons that may evolve in a correlated manner.

BUSTED analysis indicated episodic positive selection on the VP1 gene (*p* = 2.8 × 10^−5^). MEME identified 13 positively selected codons (Figure 7a) (positions 22, 24, 25, 98, 110, 111, 142, 143, 153, 160,161,174 and 202; *p* < 0.05). In contrast, the FEL method did not detect any sites that met the strict significance threshold. However, two codons (positions 44 and 161) showed marginal signals with *p*-values slightly above 0.05, suggesting weak or lineage-specific selective pressures that did not reach statistical significance under FEL. Bayesian Graphical Model (BGM) analysis identified two pairs of co-evolving codon sites within the VP1 coding region (Figure 7b). Specifically, codons 108–212 exhibited strong co-evolutionary linkage with a high posterior probability for bidirectional dependence (*P*[108↔212] = 0.95). Similarly, codons 113–177 showed evidence of co-evolution, supported by a posterior probability of 0.93 for bidirectional association.

## 4. Discussion

This study presents a comprehensive phylogenetic and evolutionary analysis of the SAT3 serotype of foot-and-mouth disease virus (FMDV), based on 81 full-length VP1 gene sequences collected from seven countries over five decades. Our findings offer new insights into the evolutionary dynamics, spatial diffusion, host transition, and selection pressures shaping the diversification of SAT3 in southern Africa. By integrating molecular phylogenetics with host ecological context, this study highlights the pivotal role of wildlife–livestock interfaces in shaping the evolutionary and epidemiological landscape of SAT3.

The likelihood mapping results revealed that the VP1 dataset carries a moderate to high phylogenetic signal, suitable for robust evolutionary inference. Phylogenetic reconstruction identified seven well-supported topotypes of SAT3 in our ML tree, which contrasts with the six topotypes previously proposed by Bastos [37] based on a broader temporal and geographic dataset. However, the BEAST-based time-calibrated phylogeny ultimately supported only five distinct topotype clusters. This discrepancy can largely be attributed to the more stringent sequence selection criteria adopted in the present study, including spatiotemporal filtering and the exclusion of sequences exhibiting aberrant temporal signals. Such an approach effectively eliminated redundant or potentially recombinant strains, thereby enhancing phylogenetic resolution while producing a more conservative and reliable topotype structure.

The presence of multiple co-circulating topotypes in countries such as South Africa, Zimbabwe, and Zambia suggests long-term endemic circulation and repeated viral introductions, likely mediated through wildlife reservoirs and animal movement [12,38]. In contrast, the more restricted topotype profiles observed in Botswana and Malawi may reflect either recent introductions or regional under-sampling. Notably, sequences from Uganda and Mozambique were excluded from our BEAST analyses due to the presence of negative temporal signals. For Uganda, previous studies have demonstrated that SAT3 isolates (e.g., SAT 3/UGA/1/13) exhibit highly mosaic genome structures with evidence of multiple recombination events, particularly involving non-capsid regions, which complicates their phylogenetic placement and leads to aberrant clock-like behavior [39]. In contrast, the phylogenetic status of the Mozambique sequence remains uncertain; while recombination cannot be ruled out, current data are insufficient to provide a definitive explanation. Further full-genome analyses will be required to clarify whether these Mozambican isolates represent genuine recombinant lineages or under-sampled indigenous variants.

The seven identified topotypes exhibited a clear pattern of geographic and host-associated differentiation. Topotypes I, II and III are distributed in both African buffalo and cattle, revealing a well-supported history of cross-species transmission. In contrast, topotype IV is restricted to African buffalo, potentially reflecting an earlier stage of viral maintenance confined to a “wildlife-only circulation” cycle. This divergence in host association reinforces the central role of wildlife reservoirs in maintaining viral diversity and transmission potential, and provides empirical evidence for identifying high-risk wildlife–livestock interfaces. Pairwise genetic distance analysis confirmed high divergence between certain topotypes (e.g., topotypes III and IV), while others, such as topotypes VI–III, shared greater similarity, potentially indicating more recent common ancestry or gene flow across regions [37].

The estimated substitution rate under the relaxed molecular clock model (3.709 × 10^−3^ substitutions/site/year) is consistent with previous estimates for SAT serotypes, including SAT1 and SAT2, which typically range between 7 × 10^−4^ and 9 × 10^−3^ substitutions/site/year [17,40,41]. These values support the conclusion that FMDV circulating in natural wildlife reservoirs or under endemic conditions evolves at a moderate but relatively stable rate.

The time to the most recent common ancestor (TMRCA) for SAT3 was inferred to be around 1875 (95% HPD: 1815–1921), which aligns with documented historical descriptions of foot-and-mouth disease-like symptoms in southern Africa during the mid- to late-19th century [42]. Demographic reconstruction using a Bayesian Skyline Plot revealed complex fluctuations in viral population size, with expansion peaking around 1985, followed by a sharp decline around 1990. These trends are likely linked to ecological and anthropogenic factors, including changes in livestock density, wildlife–livestock interface pressures, and the implementation of vaccination programs during the late 20th century [43].

The transmission network inferred from discrete phylogeographic modeling suggests a highly structured pattern of SAT3 spread in southern Africa, with Zimbabwe acting as central hubs. The identification of very strong support (BF > 100) for the Zimbabwe → South Africa transition aligns with known patterns of livestock movement and porous regional borders. Previous studies have similarly implicated these countries in the early spread of other SAT serotypes [11,44]. The inferred transmission routes and posterior probability distributions also reveal a directionally asymmetric diffusion pattern, with lower support for pathways entering countries such as Malawi and Zambia. Importantly, the posterior probabilities associated with each route support the statistical robustness of the inferred pathways [45]. We acknowledge an inherent limitation of relying on the GenBank “country” identifier as the geographic state in discrete phylogeographic analyses. High-resolution collection metadata are often unavailable or inconsistently reported; consequently, national-level state assignment may misrepresent the actual place of sampling or infection, particularly for specimens collected near international borders, moved through animal markets, or processed in laboratories located in adjacent countries. Because the model treats “country” as the unit of diffusion, such classification can inflate apparent transboundary transitions or obscure subnational circulation, thereby affecting estimates of pathway strength and directionality. In this light, our results should be viewed as broad-scale summaries of movement rather than precise reconstructions of individual border-crossing events.

Our study highlights the critical role of African buffalo as a reservoir for the SAT3 serotype of foot-and-mouth disease virus (FMDV), consistent with previous research. African buffalo are the only wildlife species proven to maintain SAT viruses asymptomatically for extended periods, sometimes for years [46,47], and are recognized as significant reservoirs in southern Africa [11,48]. They not only carry infection subclinically but also contribute to onward transmission to both wild and domestic species [49,50]. Using the updated dataset (57 *Syncerus caffer* and 18 *Bos taurus* VP1 sequences), our host-transition analysis again identified buffalo as the dominant source of spillover into cattle, with strong Bayes factor support for the African buffalo→cattle direction, reinforcing the central role of African buffalo in SAT3 maintenance and transmission. Reciprocal transitions between African buffalo and other wildlife species, as well as transitions within buffalo populations, further underline the complex wildlife [51].

Against this backdrop, the wildlife–livestock interface in southern Africa extends beyond buffalo and cattle. Notably, impala (*Aepyceros melampus*) are recognized SAT-susceptible interface hosts in surveillance settings; however, no impala VP1 sequences were available in our SAT3 dataset [52]. As a result, we could not formally reconstruct African buffalo ↔ impala ↔ cattle directionality or rates for SAT3 within our phylogeographic framework. This constitutes an evidence gap at the interface: ecological plausibility for impala involvement is high, but the direction and magnitude of SAT3 exchange involving impala remain unresolved here and warrant targeted sequencing.

Notwithstanding the strengthened evidence, we note that the cattle sample remains comparatively limited relative to African buffalo. This imbalance inevitably constrains the precision and robustness of cross-species transition inference and could influence estimated transition frequencies. Accordingly, while the directionality (African buffalo → cattle) remains strongly supported by Bayes factors, the magnitude and epidemiological generalizability of this signal should be interpreted with caution.

Taken together, these results argue for a balanced management stance: prioritize interventions at African buffalo–cattle contact points (e.g., shared grazing and water points, fence integrity, grazing logistics) while explicitly planning to incorporate impala into risk assessment as molecular data become available. Against this epidemiological backdrop, more refined management strategies are needed to avoid unnecessarily restricting wildlife conservation and commercial development, particularly in sub-Saharan regions where wildlife constitutes an important livelihood and economic resource [48]. There is a need for a better understanding of the role that different species of free-ranging wildlife play in the epidemiology of foot-and-mouth disease (FMD), so that more effective FMD management strategies and policies can be developed, particularly in southern Africa [48]. While discrete phylogeography infers cross-border diffusion among SAT3 lineages, these do not necessarily reflect direct livestock movements. Given SAT3’s limited and infrequent occurrence in cattle, most incursions likely represent local spillovers from endemically infected African buffalo populations and shared transfrontier buffalo metapopulations (e.g., Gonarezhou–Kruger, Hwange–eastern Zambia). Accordingly, our diffusion results are best interpreted as genealogical connectivity within wildlife-maintained lineages, with only sporadic buffalo-to-cattle transmission at the wildlife–livestock interface. Effective FMDV control must balance conservation, sustainable development, and disease prevention by focusing interventions at high-risk buffalo–cattle contact points (e.g., shared grazing and water sources, fence integrity, grazing logistics), while explicitly incorporating impala into risk assessment and surveillance design as molecular data become available. Integrating these actions with spatial risk mapping, vaccination planning for cattle, and bias-aware inference will help reduce cross-species transmission risk while maintaining the ecological and economic value of wildlife in endemic systems [53].

In contrast to SAT3, other FMDV serotypes display distinct phylodynamic signatures. First, evolutionary rates differ markedly: in India (1964–2012), serotype Asia1 shows a VP1 rate of ~5.87 × 10^−3^ substitutions/site/year (95% HPD ≈ 4.61–7.26 × 10^−3^) [54], generally faster than our SAT3 estimate, whereas East African SAT1 evolves more slowly at ~1.30 × 10^−3^ substitutions/site/year [17]. Second, spatial structure and lineage turnover also diverge: serotype O often exhibits multicentric, highly connected co-circulation—e.g., in India (2018–2022) the O/ME-SA/Ind2001e [55], Cluster-2018 and PanAsia-2/ANT-10 lineages circulated in parallel—while Nigerian data document both EA-3 and WA topotypes with evidence of east-to-west movements and multiple introductions [56]. Third, along the host axis, SAT serotypes (especially SAT1–3) remain tightly linked to African buffalo reservoirs with repeatedly documented buffalo-to-cattle transmission, whereas O and A spread is more often dominated by livestock movement and trade networks [56].

The VP1 gene is a critical component of the FMDV capsid, responsible for antigenicity and host cell receptor binding. It plays a pivotal role in virus–host interactions by interacting with integrin receptors on host cell surfaces, facilitating viral entry [57]. Our molecular selection analyses provide compelling evidence that the VP1 gene of the SAT3 serotype is under episodic diversifying selection, indicating adaptive pressures likely driven by host immune response or ecological shifts. The BUSTED framework detected significant episodic positive selection acting on at least one branch of the phylogeny (*p* = 2.8 × 10^−5^), a pattern consistent with previous findings that capsid proteins of FMDV often undergo topotype-specific adaptation due to immune selection or host switching [58,59].

The MEME method identified 13 codon sites (positions 22, 24, 25, 98, 110, 111, 142, 143, 153, 160, 161, 174 and 202) evolving under positive selection. Several of these positions (e.g., 142–161) lie within or near the G-H loop, the major immunogenic region of VP1 known to engage neutralizing antibodies and influence antigenicity [60]. Changes in this region may reflect antigenic drift facilitating immune evasion, a phenomenon frequently documented in SAT-type FMD viruses [61,62].

Our updated analysis identifies two co-evolving codon pairs within VP1—108–212 and 113–177. Although these sites are separated along the primary sequence and lie outside the canonical RGD-bearing G–H loop, their concerted behavior points to long-range epistatic constraints shaped by the three-dimensional architecture of the capsid. Residues 108/113 occupy the mid-N-terminal sector of VP1, whereas 177/212 lie toward the C-terminal region, a zone implicated in capsid stability and uncoating dynamics [63]. A plausible interpretation is that compensatory substitutions across these N-to-C sectors could allosterically tune epitope exposure and receptor-engagement geometry, thus maintaining particle integrity under immune pressure [37,63,64]. Mapping these residues onto a VP1 structural model and testing targeted mutants would clarify whether the paired sites approach each other in the assembled virion (e.g., across inter-protomer interfaces) and quantify their roles in stability and uncoating.

Taken together, these findings suggest that adaptive and co-adaptive pressures act on both antigenic and structural domains of the VP1 gene. This highlights the importance of monitoring evolution not only in surface-exposed epitopes but also in structurally constrained regions, which may contribute to the long-term fitness and host range of FMDV SAT3. These co-evolving residues may serve as informative targets for future vaccine design or diagnostic assay development.

## 5. Conclusions

Taken together, this study provides an integrated view of SAT3’s genetic diversity, evolutionary history, and transmission dynamics in southern Africa. The data highlight key roles for wildlife hosts, particularly *Syncerus caffer*, and transboundary viral movement in shaping the current SAT3 landscape. Insights from this study can support region-specific surveillance strategies, risk modeling, and potentially inform next-generation vaccine design by identifying evolutionary hotspots and host-associated transmission patterns. Understanding the host ecology and wildlife–livestock interface dynamics of SAT3 is essential to elucidate its evolutionary persistence and inform region-specific control strategies.

## Figures and Tables

**Table 1:** Geographical and host distribution of the seven topotypes of FMDV SAT3 identified in this study.

Topotype	Country	Host	Number of Sequences
I	South Africa, Zimbabwe	African buffalo, Cattle	34
II	Zimbabwe, Botswana, Zambia	African buffalo, Cattle	16
III	Zimbabwe; Malawi	African buffalo Cattle	21
IV	Zambia	African buffalo	3
V	Uganda	African buffalo	3
VI	Zimbabwe	African buffalo	1
VII	Mozambique	African buffalo	3

**Table 2:** Host-to-host transition rates of FMDV SAT3 inferred by Bayesian phylogeographic analysis.

From	To	Rate Mean (/Year) (ESS)	Bayes Factor	Posterior	Significance
Cattle *Bos taurus*	African buffalo *Syncerus caffer*	0.871 (9001)	0.034	0.16	No (BF < 3)
African buffalo *Syncerus caffer*	Cattle *Bos taurus*	1.08 (9001)	1631.09	1	Yes (BF > 3)

## Data Availability

For access to the information provided in this study, please contact the corresponding author upon request.

## Supplementary Materials

The following supporting information can be downloaded at: https://www.mdpi.com/article/10.3390/v17121641/s1, Supplementary Table S1. List of FMD SAT3 VP1 gene sequences by country and year used in the present study.

## References

[1] Chase-Topping M.E., Handel I., Bankowski B.M., Juleff N.D., Gibson D., Cox S.J., Windsor M.A., Reid E., Doel C., Howey R. (2013). Understanding foot-and-mouth disease virus transmission biology: Identification of the indicators of infectiousness. Vet. Res..

[2] Cockcroft P.D. (2015). Bovine Medicine.

[3] Zell R., Delwart E., Gorbalenya A.E., Hovi T., King A.M.Q., Knowles N.J., Lindberg A.M., Pallansch M.A., Palmenberg A.C., Reuter G. (2017). ICTV Virus Taxonomy Profile: Picornaviridae. J. Gen. Virol..

[4] Knowles N.J., Samuel A.R. (2003). Molecular epidemiology of foot-and-mouth disease virus. Virus Res..

[5] Rweyemamu M., Roeder P., Mackay D., Sumption K., Brownlie J., Leforban Y., Valarcher J.F., Knowles N.J., Saraiva V. (2008). Epidemiological patterns of foot-and-mouth disease worldwide. Transbound. Emerg. Dis..

[6] Knight-Jones T.J.D., Rushton J. (2013). The economic impacts of foot and mouth disease–What are they, how big are they and where do they occur?. Prev. Vet. Med..

[7] Brooksby J.B. (1958). The virus of foot-and-mouth disease. Adv. Virus Res..

[8] Brown F. (2003). The history of research in foot-and-mouth disease. Virus Res..

[9] Maake L., Harvey W.T., Rotherham L., Opperman P., Theron J., Reeve R., Maree F.F. (2020). Genetic Basis of Antigenic Variation of SAT3 Foot-And-Mouth Disease Viruses in Southern Africa. Front. Vet. Sci..

[10] Dyason E. (2010). Summary of foot-and-mouth disease outbreaks reported in and around the Kruger National Park, South Africa, between 1970 and 2009. J. S. Afr. Vet. Assoc..

[11] Bastos A.D., Boshoff C.I., Keet D.F., Bengis R.G., Thomson G.R. (2000). Natural transmission of foot-and-mouth disease virus between African buffalo (*Syncerus caffer*) and impala (*Aepyceros melampus*) in the Kruger National Park, South Africa. Epidemiol. Infect..

[12] Brito B.P., Jori F., Dwarka R., Maree F.F., Heath L., Perez A.M. (2016). Transmission of Foot-and-Mouth Disease SAT2 Viruses at the Wildlife-Livestock Interface of Two Major Transfrontier Conservation Areas in Southern Africa. Front. Microbiol..

[13] Mason P.W., Grubman M.J., Baxt B. (2003). Molecular basis of pathogenesis of FMDV. Virus Res..

[14] Belsham G.J. (1993). Distinctive features of foot-and-mouth disease virus, a member of the picornavirus family; aspects of virus protein synthesis, protein processing and structure. Prog. Biophys. Mol. Biol..

[15] Bachanek-Bankowska K., Di Nardo A., Wadsworth J., Mioulet V., Pezzoni G., Grazioli S., Brocchi E., Kafle S.C., Hettiarachchi R., Kumarawadu P.L. (2018). Reconstructing the evolutionary history of pandemic foot-and-mouth disease viruses: The impact of recombination within the emerging O/ME-SA/Ind-2001 lineage. Sci. Rep..

[16] Le V.P., Vu T.T., Duong H.Q., Than V.T., Song D. (2016). Evolutionary phylodynamics of foot-and-mouth disease virus serotypes O and A circulating in Vietnam. BMC Vet. Res..

[17] Sangula A.K., Belsham G.J., Muwanika V.B., Heller R., Balinda S.N., Masembe C., Siegismund H.R. (2010). Evolutionary analysis of foot-and-mouth disease virus serotype SAT 1 isolates from east Africa suggests two independent introductions from southern Africa. BMC Evol. Biol..

[18] Katoh K., Standley D.M. (2013). MAFFT multiple sequence alignment software version 7: Improvements in performance and usability. Mol. Biol. Evol..

[19] Martin D.P., Murrell B., Golden M., Khoosal A., Muhire B. (2015). RDP4: Detection and analysis of recombination patterns in virus genomes. Virus Evol..

[20] Xia X. (2018). DAMBE7: New and Improved Tools for Data Analysis in Molecular Biology and Evolution. Mol. Biol. Evol..

[21] Kalyaanamoorthy S., Minh B.Q., Wong T.K.F., von Haeseler A., Jermiin L.S. (2017). ModelFinder: Fast model selection for accurate phylogenetic estimates. Nat. Methods.

[22] Schmidt H.A., Strimmer K., Vingron M., von Haeseler A. (2002). TREE-PUZZLE: Maximum likelihood phylogenetic analysis using quartets and parallel computing. Bioinformatics.

[23] Nguyen L.T., Schmidt H.A., von Haeseler A., Minh B.Q. (2015). IQ-TREE: A fast and effective stochastic algorithm for estimating maximum-likelihood phylogenies. Mol. Biol. Evol..

[24] Kumar S., Stecher G., Tamura K. (2016). MEGA7: Molecular Evolutionary Genetics Analysis Version 7.0 for Bigger Datasets. Mol. Biol. Evol..

[25] Susko E., Inagaki Y., Roger A.J. (2004). On inconsistency of the neighbor-joining, least squares, and minimum evolution estimation when substitution processes are incorrectly modeled. Mol. Biol. Evol..

[26] Rambaut A., Lam T.T., Max Carvalho L., Pybus O.G. (2016). Exploring the temporal structure of heterochronous sequences using TempEst (formerly Path-O-Gen). Virus Evol..

[27] Suchard M.A., Lemey P., Baele G., Ayres D.L., Drummond A.J., Rambaut A. (2018). Bayesian phylogenetic and phylodynamic data integration using BEAST 1.10. Virus Evol..

[28] Ayres D.L., Darling A., Zwickl D.J., Beerli P., Holder M.T., Lewis P.O., Huelsenbeck J.P., Ronquist F., Swofford D.L., Cummings M.P. (2012). BEAGLE: An application programming interface and high-performance computing library for statistical phylogenetics. Syst. Biol..

[29] Xie W., Lewis P.O., Fan Y., Kuo L., Chen M.H. (2011). Improving marginal likelihood estimation for Bayesian phylogenetic model selection. Syst. Biol..

[30] Fourment M., Magee A.F., Whidden C., Bilge A., Matsen F.A., Minin V.N. (2020). 19 Dubious Ways to Compute the Marginal Likelihood of a Phylogenetic Tree Topology. Syst. Biol..

[31] Rambaut A., Drummond A.J., Xie D., Baele G., Suchard M.A., Susko E. (2018). Posterior Summarization in Bayesian Phylogenetics Using Tracer 1.7. Syst. Biol..

[32] Nahata K.D., Bielejec F., Monetta J., Dellicour S., Rambaut A., Suchard M.A., Baele G., Lemey P. (2022). SPREAD 4: Online visualisation of pathogen phylogeographic reconstructions. Virus Evol..

[33] Delport W., Poon A.F., Frost S.D., Kosakovsky Pond S.L. (2010). Datamonkey 2010: A suite of phylogenetic analysis tools for evolutionary biology. Bioinformatics.

[34] Kosakovsky Pond S.L., Frost S.D.W. (2005). Not So Different After All: A Comparison of Methods for Detecting Amino Acid Sites Under Selection. Mol. Biol. Evol..

[35] Murrell B., Moola S., Mabona A., Weighill T., Sheward D., Kosakovsky Pond S.L., Scheffler K. (2013). FUBAR: A Fast, Unconstrained Bayesian AppRoximation for Inferring Selection. Mol. Biol. Evol..

[36] Ke G.M., Ho C.H., Chiang M.J., Sanno-Duanda B., Chung C.S., Lin M.Y., Shi Y.Y., Yang M.H., Tyan Y.C., Liao P.C. (2015). Phylodynamic analysis of the canine distemper virus hemagglutinin gene. BMC Vet. Res..

[37] Bastos A.D., Anderson E.C., Bengis R.G., Keet D.F., Winterbach H.K., Thomson G.R. (2003). Molecular epidemiology of SAT3-type foot-and-mouth disease. Virus Genes.

[38] Vosloo W., Boshoff K., Dwarka R., Bastos A. (2002). The possible role that buffalo played in the recent outbreaks of foot-and-mouth disease in South Africa. Ann. N. Y. Acad. Sci..

[39] Lasecka-Dykes L., Wright C.F., Di Nardo A., Logan G., Mioulet V., Jackson T., Tuthill T.J., Knowles N.J., King D.P. (2018). Full Genome Sequencing Reveals New Southern African Territories Genotypes Bringing Us Closer to Understanding True Variability of Foot-and-Mouth Disease Virus in Africa. Viruses.

[40] Hall M.D., Knowles N.J., Wadsworth J., Rambaut A., Woolhouse M.E. (2013). Reconstructing geographical movements and host species transitions of foot-and-mouth disease virus serotype SAT 2. mBio.

[41] Lycett S., Tanya V.N., Hall M., King D.P., Mazeri S., Mioulet V., Knowles N.J., Wadsworth J., Bachanek-Bankowska K., Ngu Ngwa V. (2019). The evolution and phylodynamics of serotype A and SAT2 foot-and-mouth disease viruses in endemic regions of Africa. Sci. Rep..

[42] Thomson G.R. (1995). Overview of foot and mouth disease in southern Africa. Rev. Sci. Tech..

[43] Casey-Bryars M., Reeve R., Bastola U., Knowles N.J., Auty H., Bachanek-Bankowska K., Fowler V.L., Fyumagwa R., Kazwala R., Kibona T. (2018). Waves of endemic foot-and-mouth disease in eastern Africa suggest feasibility of proactive vaccination approaches. Nat. Ecol. Evol..

[44] Bronsvoort B.M., Radford A.D., Tanya V.N., Nfon C., Kitching R.P., Morgan K.L. (2004). Molecular epidemiology of foot-and-mouth disease viruses in the Adamawa province of Cameroon. J. Clin. Microbiol..

[45] Calkins C.M., Scasta J.D. (2020). Transboundary Animal Diseases (TADs) affecting domestic and wild African ungulates: African swine fever, foot and mouth disease, Rift Valley fever (1996–2018). Res. Vet. Sci..

[46] Omondi G.P., Gakuya F., Arzt J., Sangula A., Hartwig E., Pauszek S., Smoliga G., Brito B., Perez A., Obanda V. (2020). The role of African buffalo in the epidemiology of foot-and-mouth disease in sympatric cattle and buffalo populations in Kenya. Transbound. Emerg. Dis..

[47] Lillian S. (2021). African Buffalo persistently exposed to contagious foot and mouth diseases. Nat. Afr..

[48] Vosloo W., Swanepoel S.P., Bauman M., Botha B., Esterhuysen J.J., Boshoff C.I., Keet D.F., Dekker A. (2011). Experimental infection of giraffe (*Giraffa camelopardalis*) with SAT-1 and SAT-2 foot-and-mouth disease virus. Transbound. Emerg. Dis..

[49] Sirdar M.M., Fosgate G.T., Blignaut B., Mampane L.R., Rikhotso O.B., Du Plessis B., Gummow B. (2021). Spatial distribution of foot-and-mouth disease (FMD) outbreaks in South Africa (2005–2016). Trop. Anim. Health Prod..

[50] Jori F., Etter E. (2016). Transmission of foot and mouth disease at the wildlife/livestock interface of the Kruger National Park, South Africa: Can the risk be mitigated?. Prev. Vet. Med..

[51] Mastroeni P., Grant A., Restif O., Maskell D. (2009). A dynamic view of the spread and intracellular distribution of Salmonella enterica. Nat. Rev. Microbiol..

[52] Vosloo W., Thompson P.N., Botha B., Bengis R.G., Thomson G.R. (2009). Longitudinal study to investigate the role of impala (*Aepyceros melampus*) in foot-and-mouth disease maintenance in the Kruger National Park, South Africa. Transbound. Emerg. Dis..

[53] Mashinagu M.M., Wambura P.N., King D.P., Paton D.J., Maree F., Kimera S.I., Rweyemamu M.M., Kasanga C.J., Samrat S. (2024). Challenges of Controlling Foot-and-Mouth Disease in Pastoral Settings in Africa. Transbound. Emerg. Dis..

[54] Subramaniam S., Mohapatra J.K., Sharma G.K., Das B., Dash B.B., Sanyal A., Pattnaik B. (2013). Phylogeny and genetic diversity of foot and mouth disease virus serotype Asia1 in India during 1964–2012. Vet. Microbiol..

[55] Dahiya S.S., Subramaniam S., Mohapatra J.K., Rout M., Biswal J.K., Giri P., Nayak V., Singh R.P. (2023). Foot-and-Mouth Disease Virus Serotype O Exhibits Phenomenal Genetic Lineage Diversity in India during 2018–2022. Viruses.

[56] Ularamu H.G., Ibu J.O., Wood B.A., Abenga J.N., Lazarus D.D., Wungak Y.S., Knowles N.J., Wadsworth J., Mioulet V., King D.P. (2017). Characterization of Foot-and-Mouth Disease Viruses Collected in Nigeria Between 2007 and 2014: Evidence for Epidemiological Links Between West and East Africa. Transbound. Emerg. Dis..

[57] Jackson T., King A.M., Stuart D.I., Fry E. (2003). Structure and receptor binding. Virus Res..

[58] Haydon D.T., Bastos A.D., Knowles N.J., Samuel A.R. (2001). Evidence for positive selection in foot-and-mouth disease virus capsid genes from field isolates. Genetics.

[59] Martinez M.A., Dopazo J., Hernandez J., Mateu M.G., Sobrino F., Domingo E., Knowles N.J. (1992). Evolution of the capsid protein genes of foot-and-mouth disease virus: Antigenic variation without accumulation of amino acid substitutions over six decades. J. Virol..

[60] Carrillo C., Tulman E.R., Delhon G., Lu Z., Carreno A., Vagnozzi A., Kutish G.F., Rock D.L. (2005). Comparative genomics of foot-and-mouth disease virus. J. Virol..

[61] Tully D.C., Fares M.A. (2006). Unravelling Selection Shifts among Foot-and-Mouth Disease virus (FMDV) Serotypes. Evol. Bioinform..

[62] Tully D.C., Fares M.A. (2009). Shifts in the selection-drift balance drive the evolution and epidemiology of foot-and-mouth disease virus. J. Virol..

[63] Logan D., Abu-Ghazaleh R., Blakemore W., Curry S., Jackson T., King A., Lea S., Lewis R., Newman J., Parry N. (1993). Structure of a major immunogenic site on foot-and-mouth disease virus. Nature.

[64] Acharya R., Fry E., Stuart D., Fox G., Rowlands D., Brown F. (1989). The three-dimensional structure of foot-and-mouth disease virus at 2.9 A resolution. Nature.

